# Screw Stress Distribution in a Clavicle Fracture with Plate Fixation: A Finite Element Analysis

**DOI:** 10.3390/bioengineering10121402

**Published:** 2023-12-07

**Authors:** Angelo Alito, Domenico Fenga, Giada Tropeano, Demetrio Milardi, Danilo Leonetti, Alba Migliorato, Adriana Tisano, Danilo D’Andrea, Vincenzo Filardi

**Affiliations:** 1 Department of Biomedical, Dental Sciences and Morphological and Functional Images, University of Messina, 98125 Messina, Italy; alitoa@unime.it (A.A.); giadatropeano@hotmail.it (G.T.); dmilardi@unime.it (D.M.); dleonetti@unime.it (D.L.); alba.migliorato@unime.it (A.M.); 2 Department of Orthopaedics and Traumatology, University Hospital A.O.U. “G. Martino”, 98125 Messina, Italy; dfenga@gmail.com; 3 Department of Clinical and Experimental Medicine, University of Messina, 98125 Messina, Italy; atisano@unime.it; 4 Department of Engineering, University of Messina, 98158 Messina, Italy; danilo.dandrea@unime.it; 5 D.A. Scientific Research and Internationalization, University of Messina, 98121 Messina, Italy

**Keywords:** clavicle plate, fractured clavicle, finite element analysis, finite element modeling, screw stress distribution, stress analysis

## Abstract

Clavicle midshaft fractures are mostly treated surgically by open internal reduction with a superior or anteroinferior plate and screws or by intramedullary nailing. Screw positioning plays a critical role in determining the stress distribution. There is a lack of data on the screw position and the appropriate number of cortices required for plate fixation. The aim of this study is to evaluate the mechanical behavior of an anterior plate implanted in a fractured bone subjected to 120° of lateral elevation compared to a healthy clavicle using numerical simulations. Contact forces and moments used were obtained from literature data and applied to the healthy and fractured finite element models. Stresses of about 9 MPa were found on the healthy clavicle, while values of about 15 MPa were calculated on the plate of the fractured one; these stress peaks were reached at about 30° and 70° of elevation when the stress shielding on the clavicle sums all the three components of the solicitation: compression, flexion, and torsion. The stress distribution in a clavicle fracture stabilized with plates and screws is influenced by several factors, including the plate’s position and design, the type of screw, and the biomechanical forces applied during movements.

## 1. Introduction

**Figure 1:**
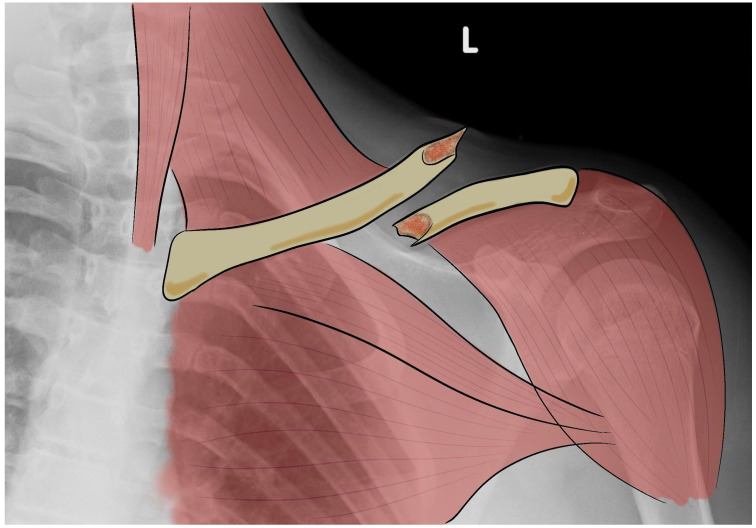
Representation of an X-ray image with drawing of a clavicle fracture. L: left side.

In the field of orthopedic surgery, clavicle fractures are a common injury that often requires surgical intervention for proper healing [1]. The clavicle has three parts: body (or diaphysis), sternal end (or medial epiphysis), and acromial end (or lateral epiphysis) [2,3]. The biomechanics of a healthy clavicle involve the absorption and distribution of stress during bending and compressive loads to ensure optimal function and stability [4]. It is involved in scapulothoracic kinematic with the acromioclavicular (AC) and sternoclavicular (SC) joints [5]. At the SC joint, scapulothoracic movements have been shown to cause slight rotation of the clavicle relative to the thorax, which occurs more frequently at the SC joint than at the AC joint [5]. Shrugging resulted in a very large increase in clavicle elevation of 25 degrees [6]. Clavicle fractures are one of the most common traumatic injuries in adults, accounting for approximately 5% of all fractures, and half of all of these are shoulder girdle fractures [7,8]. Most fractures involve the middle third of the clavicle, which, because of its thin structure and subcutaneous location, is the only area not protected or reinforced by muscle and ligament attachments (Figure 1) [9].

To understand the fracture mechanism, it is important to consider the deformation forces acting on this segment [3,10,11], which are usually the result of high-energy blunt trauma to the lateral shoulder, with a force greater than the elastic limit of the bone [12]. In other cases, the trauma occurs by transfer following a fall on an outstretched arm, which disperses the traumatic force rather than concentrating it directly on the clavicle [13]. Sports-related activities such as cycling or contact sports have also been identified as common causes of clavicle fractures [14].

Clinically, clavicle fractures are usually visible on initial examination as a visible and/or palpable deformity with ecchymosis and an underlying sensation of emptiness on palpation, and patients report specific pain at the fracture site [13,15]. The deforming muscle forces produce a characteristic clinical appearance of shoulder drooping, scapular internal rotation, and shoulder shortening [15].

Although numerous classification systems for clavicle fractures have been described (AO, Neer, Craig, Robinson), the simplest and most widely used is the Allman classification, which divides clavicle fractures into three groups according to their anatomical location in relation to the attachments of the sternocleidomastoid and trapezius muscles [16,17]. In the Allman classification, the middle third of the clavicle represents group I of clavicle fractures with a frequency of 80% compared to the lateral (group II) and medial (group III) parts (15% and 5%, respectively) [16,17]. Each type is divided into three subgroups according to the direction of displacement: A, minimal displacement; B, displacement with overlapping fragments; and C, displacement with complete separation [16]. This classification can help determine the appropriate management approach. In fact, Type I fractures will often recover well with a conservative approach, whereas Type II and Type III may require more caution, especially if there is significant displacement [18].

Diagnostic methods are required to investigate the type of fracture for more appropriate management, consisting of two radiographs taken in the standing position—a standard anteroposterior view and a 15° cephalic oblique view to define the superior or inferior displacement of the fracture [19]. Computed tomography (CT) is not routinely performed but may be useful for medial fractures or in the presence of associated scapular or thoracic fractures [20].

Clavicle fractures can lead to complications if not treated properly, such as malunion or nonunion of the fracture, resulting in persistent pain and limited range of motion [21].

A better understanding of this type of fracture has led to improved operative management with excellent results in terms of functional outcome [22]. Most patients with compound or minimally compound fractures of the middle third of the clavicle (less than 1.5 cm shortening) can be managed non-operatively with a sling that supports the weight of the elbow, with a gradual return to motion approximately four weeks after the traumatic event [21]. As the indications for surgical treatment have expanded due to the excellent results, the effectiveness of non-surgical treatment has been questioned, with mixed results for both types of approach [21,23,24]. Surgery is indicated in cases of severe diastasis of the fragments, fractures with a third vertical fragment protruding under the skin, pseudoarthrosis, irreducible fractures after immobilization, and rapid return to work [25].

Therefore, based on the available evidence, surgical treatment, particularly plate fixation, is increasingly favored for displaced midshaft clavicle fractures in younger and active patients [26,27]. There are several reasons for this shift towards surgical treatment. Firstly, recent studies have shown that non-operative treatment of displaced midshaft clavicle fractures leads to poorer outcomes than surgical treatment [27,28]. Secondly, advances in surgical techniques and a better understanding of the fracture type have contributed to the increasing preference for surgical management [1]. In addition, plate fixation offers biomechanical advantages, including greater stiffness and flexural strength compared to intramedullary fixation [28,29].

Plate fixation also allows for smaller incisions, less soft tissue disruption, and avoids damage to the supraclavicular sensory nerves [30]. In addition, studies have shown that early surgery is preferred to delayed surgery in the management of this type of fracture. An early approach is preferred because it allows early rehabilitation and has shown favorable results in terms of functional recovery [31]. On the other hand, delayed surgery may result in prolonged pain and delayed return to function and may lead to higher rates of nonunion and symptomatic malunion [31].

**Figure 2:**
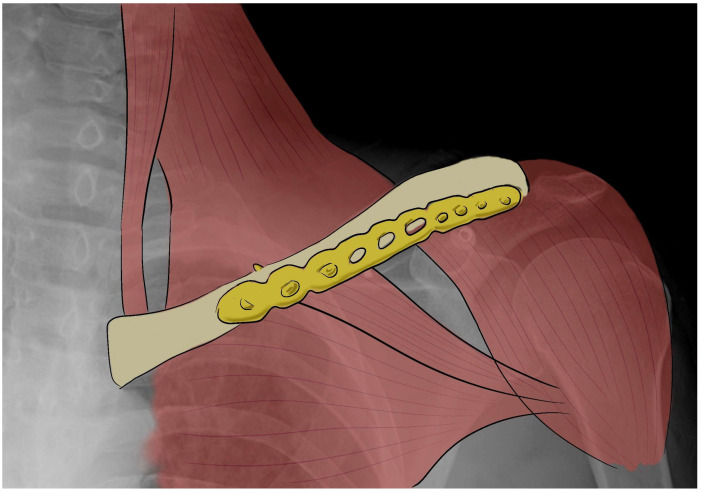
X-ray image with a draw of clavicle fracture fixed by an anteroinferior plate.

Fractures of the middle portion of the clavicle are most treated surgically by open internal reduction with a superior or anteroinferior plate and screws or by intramedullary nailing (Figure 2) [32,33,34,35].

Additionally, the positioning and placement of screws play a crucial role in determining the stress distribution in clavicle fracture fixation [36].

Rehabilitation protocols for operative and non-operative treatment of midshaft clavicle fractures differ significantly. The protocol for operative treatment typically includes early mobilization and range of motion exercises to prevent stiffness and promote healing; movement is allowed very quickly because the stability provided by plate fixation allows early function [37].

Non-operative treatment, on the other hand, usually involves immobilization with a cast to allow the fracture to heal naturally [38]. In fact, rehabilitation is often delayed, allowing the fracture to heal completely and avoid stressing the clavicle in the initial phase. This type of treatment often results in an increased rate of re-injury, a delayed recovery to daily activities, and sub-optimal function of the shoulder due to malunion and shortening of the clavicle, with consequent thoraco-scapular dyskinesia [38].

There is a lack of data on the screw position and the appropriate number of cortices required for plate fixation and on the long-term outcomes and potential complications associated with surgical treatment of displaced midshaft clavicle fractures.

The aim of this study is to estimate the stress distribution in a clavicle fracture stabilized with an anteroinferior plate and screws, compared to a healthy one, during shoulder elevation using numerical finite element (FE) analysis. The model has been developed to assess the forces involved in the biomechanics of the clavicle during shoulder elevation and the role of the stabilization plate and screws to guide the choice of the best possible treatment. Considering the multiple components involved in human movement, analyzing the forces involved in fixation devices seems to be a good way to improve their application, design, and rehabilitation approach. Furthermore, the FE model can help to analyze the stability of the fracture fixation, the degree of bone and implant stress, and the bone adaptation to the plate and screws in depth and could lead to a better implant technique to ensure biomechanical stability.

## 2. Materials and Methods

**Figure 3:**
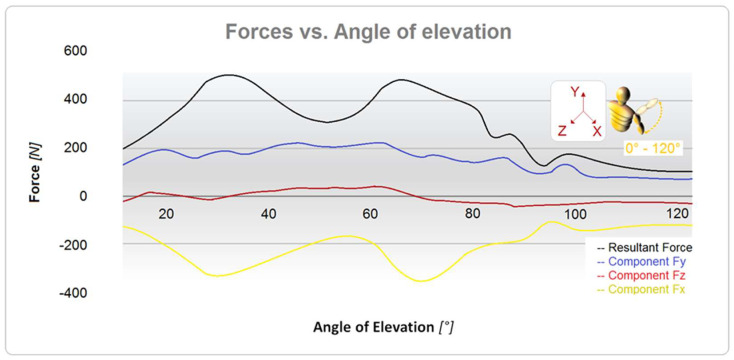
Curves of forces vs. angle of external rotation evaluated on the humerus.

**Figure 4:**
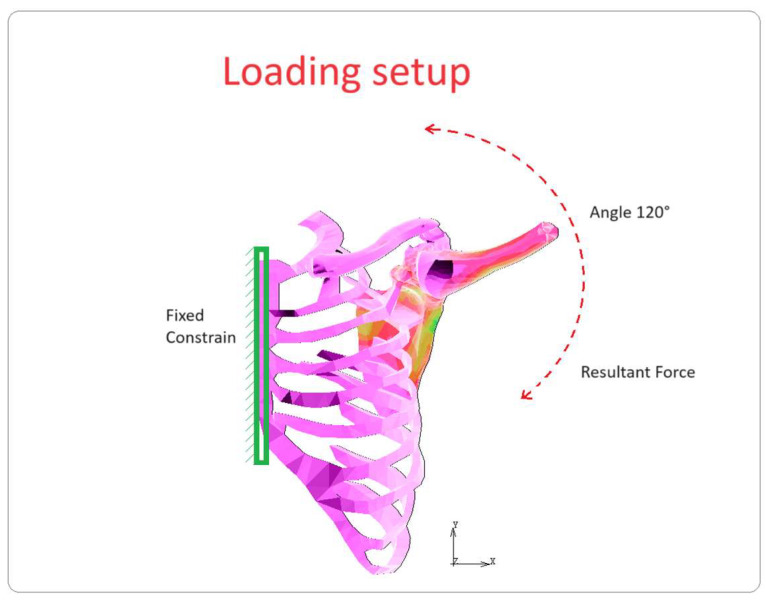
Schematic loading conditions.

In order to assess the forces applied to the shoulder joint, a three-dimensional joint model was created and loaded using data derived from a clinically validated shoulder implant (BIOMODULAR, Biomet Germany, Berlin, Germany, with six strain gauges and nine-channel telemetry). Peak resultant forces (Fp) were determined using MATLAB software (ver. R2023b), as shown in Figure 3, which shows the angle of elevation (°) vs. force (N) curves. In Figure 4, the schematic loading conditions to perform numerical analysis are represented.

Simulations were performed by imposing an elevation of the humerus from 0° to 120° along the Z-axes. An anteroinferior plate was implanted in the middle third shaft fracture (75% of clavicle fractures) [14]. Two different 3D FE numerical models of the healthy and unhealthy clavicles were created from ten-node tetrahedral elements using CT scan data, and the various parts were modeled (Table 1). The characteristics of all the bony elements (rib cage, scapula, humerus, and clavicle) were defined by choosing an elastic modulus of 17,500 MPa and a Poisson ratio of 0.35, whereas the ligaments were modeled with an elastic modulus of 128 MPa. The mechanical properties of the material have been chosen considering the maximum value of the elastic modulus, E = 210 GPa, which can be achieved by specific surface treatments of the steel and by adding elements such as Co or Mg to the alloy, as reported in the literature [39,40,41]. Future developments of this work aim to investigate different material behaviors in depth, including titanium and its alloys and screw configurations.

The model was solved using ANSYS 2022 R2 (22.2) software. The nodes of the ribcage were fixed along the axes of symmetry, and the force curves shown in Figure 4 were applied. The accuracy of the results was checked using some comparisons, and a convergence study based on the stresses in the areas of interest was required.

## 3. Results

**Figure 5:**
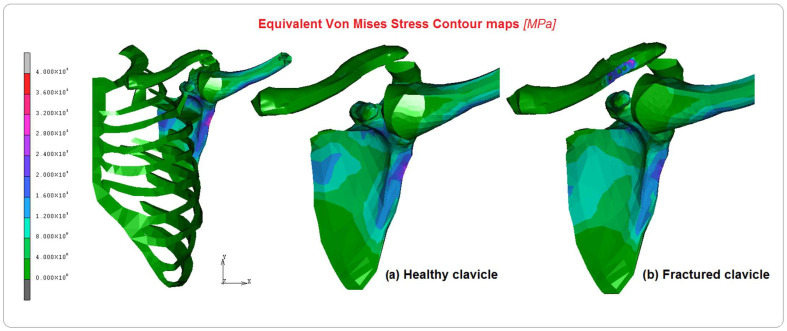
Equivalent von Mises contour maps on the FE model and comparison between (**a**) healthy clavicle and (**b**) implanted clavicle.

Stresses of about 40 MPa were found on the humerus and 30 MPa on the scapula during the elevation phase, acting at 34° and 70°, as shown in Figure 5. In Figure 5a,b, it is possible to see the different stress levels visible on the clavicle in the healthy and fractured cases, respectively. Table 2 shows the equivalent von Mises stress calculated for each part. The glenohumeral, acromioclavicular, and coracohumeral ligaments showed stresses lower than 13 MPa.

**Figure 6:**
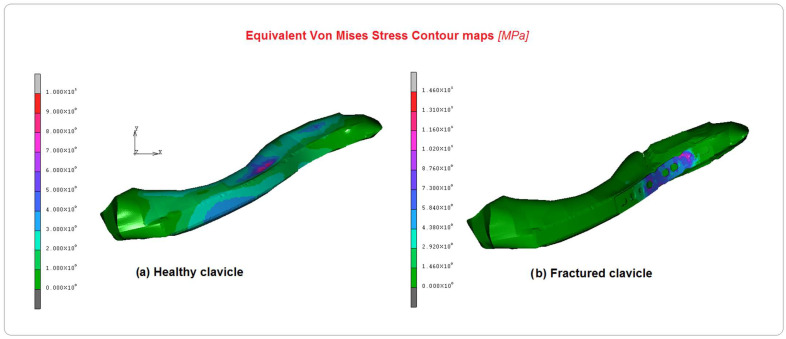
Comparison of equivalent von Mises contours between (**a**) healthy clavicle and (**b**) implanted clavicle.

**Figure 7:**
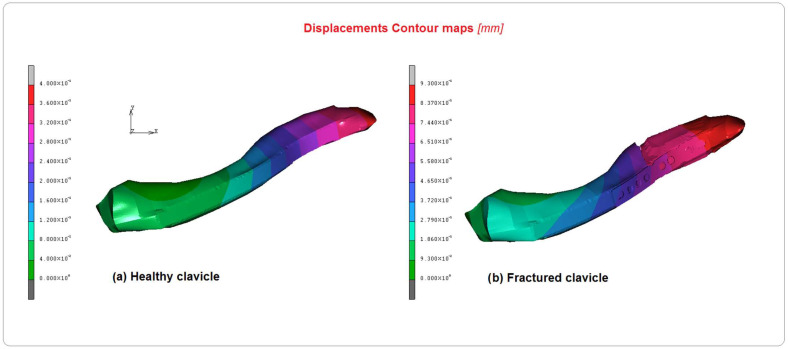
Comparison of displacement contour maps between (**a**) healthy clavicle and (**b**) implanted clavicle.

**Figure 8:**
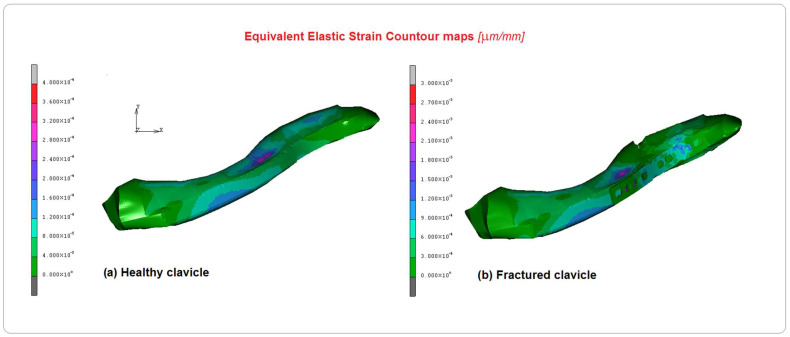
Comparison of the equivalent elastic strain contour maps between (**a**)the healthy clavicle and (**b**) the implanted clavicle.

Figure 6, Figure 7 and Figure 8 show the comparison between the healthy and implanted clavicles in terms of equivalent von Mises stress, displacements, and equivalent elastic strain. As can be seen from the analysis of Figure 6, the stress on the healthy clavicle is approximately 9 MPa, while the other one reaches approximately 15 MPa. This level of loading is completely absorbed by the screws and plate. The predominant type of solicitation is compression–flexion aging in the axial direction of the bone. Figure 7 shows the displacements recorded in the two cases. Significant differences can be argued on the healthy clavicle of about 0.4 mm and 0.93 mm on the other. This is because of the specific function of the plate, which can only be attached to the bone on one side. It is also due to the specific nature of the injury, which tends to open the two bony fragments on the opposite side.

**Figure 9:**
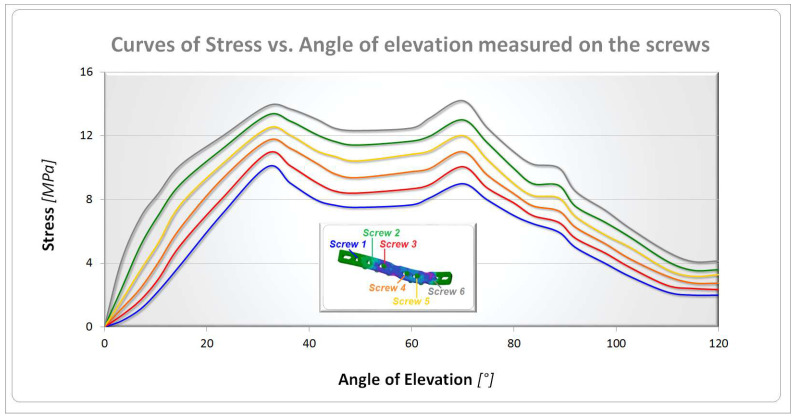
Stress vs. angle of elevation curves for screws.

Figure 8 shows the equivalent elastic strain contour maps in both cases. The results confirm a coherent behavior with the other previous results obtained for stresses and displacements; values of 0.0004 µm/mm were observed in the healthy clavicle and 0.003 mm/mm in the other. Finally, Figure 9 presents the curves of the equivalent von Mises stress versus the angle of elevation measured on the screws. As shown in the figure, the screws have been numbered from 1 to 6, and the respective curves have been plotted on the graph. As can be seen, the screw with the highest stress, approximately 14 MPa, is number 6, probably due to its proximity to the glenohumeral joint and its functional role in maintaining the two attached bony ends. The second screw to play an important role is number 2, where the stress reached a level of about 13 MPa. These results must be considered considering the peculiar kind of solicitation imposed. During its elevation, from 0° to 120°, the humerus, which is connected through the kinematic bony and tissue chain, transmits different levels of stress to the clavicle at different elevation angles. At 30° and 70°, the main stress components evaluated in screw 6 are caused by shear/compression stress-induced aging of the first clavicle stump. The stress then progressively spreads inside the stump through screws 5 and 4. Successively, the plate sustains the stress connecting the second stump and transmitting the higher part of it to screw 2. By observing the different stress components acting in equivalent von Mises stress values, evaluated during the FE analysis, the most important contribution is played by the shear solicitation derived by the connection with the plate. The load on screws 3 and 1 is gradually transferred.

## 4. Discussion

The aim of the present research was to estimate stress distribution occurring in a clavicle fracture surgically treated with a plate, compared to a healthy one, during elevation of the shoulder. Numerous previous studies have used FE analysis to gain a better understanding of the disease consequences in terms of timing, outcomes, and healthcare costs [35,42,43,44]. Several authors have demonstrated improved early functional outcomes with lower rates of nonunion and symptomatic aesthetic deformity with surgical treatment of clavicle fractures compared to non-surgical treatment [45,46]. The decision between surgical and non-surgical treatment should be based on several factors, such as the severity of the injury, the occurrence of complications, the patient’s age, and the activity level [14]. Indeed, no solid evidence exists that the long-term functional outcome of surgery is significantly superior to non-surgical treatment [21]. After a bone fracture, if surgery is required, the choice of device depends on the fracture characteristics and on the surgeon’s preference [18,47], but functional needs are one of the most important points in the choice of treatment [48,49]. Clinical implications for the choice of fixation type include consideration of the potential stress distribution and fixation failure associated with different plate designs in clavicle fracture fixation [50]. This stress concentration is due to factors such as plate fracture or deformation, excessive stress concentration, and the position of the plate and screws used to fix the fracture [51,52]. When intramedullary fixation is compared with plate and screw fixation, the latter offers a greater biomechanical advantage in terms of stiffness and bending strength [18,50]. The advantages of intramedullary fixation of clavicle fractures include a smaller incision, less soft tissue disruption, less bone prominence, and avoidance of supraclavicular sensory nerves [53].

Several designs of anatomically preformed plates are available for the fixation of clavicle fractures. Ideally, these 3.5 mm anatomically precontoured plates, which are designed to conform to the superior clavicle surface, should be universally conformable and have minimal hardware-related complications compared to standard uncontoured plates [54].

The morphological variability of the clavicle in patients makes it difficult to provide a standard precontoured implant that fits the wide range of clavicle shapes. When precontoured implants are used to fix clavicle fractures, it is often found that the plates do not conform to the clavicle anatomy [54]. The choice of an anterior plate partially reduces the risk of complications related to the superior plate and reduces anatomical variability [55].

The anterior clavicle plate helps to hold the bone fragments together as they heal, ensuring proper alignment and function of the clavicle [56].

These plates are typically thin and contoured to match the natural shape of the clavicle and are specifically designed to fit the anterior part of the clavicle, providing a secure attachment that minimizes the risk of implant migration or loosening [56].

Common materials used for clavicle plates are titanium and stainless steel. Titanium is a light, solid, and highly biocompatible material widely used in surgical implants, known for its resistance to corrosion and its compatibility with MRI scans [57]. Stainless steel is strong and durable but may not be as MRI-friendly as titanium [57].

The plate typically has multiple holes or slots to accommodate screws, which are strategically placed to allow for secure fixation along the bone, ensuring that the fragments are held together properly [56]. Some plates have locking mechanisms that allow the screws to lock in the metal plate, providing additional stability [58]. Locking screws help prevent plate loosening and maintain compression [58]. The design of the plate aims to be as low profile as possible to minimize soft tissue irritation and discomfort for the patient [56]. In addition, screws are an integral part of clavicle fracture fixation and are typically made from the same biocompatible materials as the plate [58]. This is usually used to treat high-functioning athletes or people with severe deformities [3].

In fact, in a recent systematic literature review, the authors found that in adult patients with displaced mid-third clavicle fractures, surgical treatment was associated with a greater likelihood of union at one year [59]. Overall, surgical treatment did not increase functional scores by amounts those patients were likely to consider clinically important. Considering these findings, they believe that patients can be informed that surgery for this injury can incrementally increase the likelihood of union (about 10 patients would need to undergo surgery to avoid one nonunion), but they should not expect better function than they would achieve without surgery; most patients can avoid surgery altogether with a low absolute risk of nonunion [59]. The aim is to achieve early functional recovery and reduce the amount of time spent resting or out of sport.

The current literature shows that there is currently no difference in hardware removal rates or functional outcomes when using a superior versus anterior plating technique [18].

Shoulder elevation is a common activity in everyday life that increases the tensile and compressive forces on the clavicle and can affect the stress distribution within the fixation devices in a stabilized fracture [60,61,62,63].

Our analysis showed that the stresses acting on the fractured clavicle, expressed in equivalent von Mises stress, are approximately 15 MPa, but this is supported by the fixation system in the most common type of solicitation. There is also a displacement of 0.93 mm between the ends of the bone. These results indicate that the use of an anteroinferior plate fixed with screws in clavicle fracture fixation may result in an altered stress distribution compared to a healthy clavicle. These results could provide a valuable biomechanical reference for orthopedic surgeons, highlighting the important role of screws in absorbing and distributing loading forces in clavicle fractures during shoulder elevation and suggesting that the correct number of screws plays a key role in the repair and stability of the bone ends.

Some authors had previously studied clavicle fixation with FE analysis and found that when screw holes were positioned close to the fracture site, there was a significant increase in stress on the plate and clavicle [64,65]. This indicates that screw positioning close to the fracture can have a significant effect on the stress pattern in the plate fixation system [66]. These changes in stress patterns can potentially affect the healing process, long-term fracture stability, rehabilitation program, and clinical outcomes [67].

The number and position of screws used in plate fixation can influence the rehabilitation plan. For example, using a greater number of screws may provide greater stability and allow earlier full-range mobility exercises during rehabilitation [64]. On the other hand, if only a few screws are used or if they are placed in specific positions, caution may be required during rehabilitation to avoid stressing the implant or compromising its stability [68].

In addition, one study suggested that the maximum stress in clavicle fracture plate fixation without lag screws occurs at the edge of the hole above the fracture site [69]. In fact, stress concentration occurs around the empty screw holes above the fracture site when a clavicle fracture is stabilized with plates and screws [51]. The maximum stress point is typically found at the edge of the holes, indicating that these areas are more susceptible to failure. The greater the load on the bone and the greater the risk of fracture near the plate, the fewer screws are implanted in the plate.

In addition to plate position, other important factors to consider include the reconstruction plate and bridging plate technique, which may raise the risk of plate fracture [69]. It is, therefore, important to consider the design and placement of the plate to minimize stress and potential complications due to clavicle anatomical position that can cause serious damage to valuable structures [70,71]. In addition, deep learning algorithms for radiological images could be used to improve the customization of the shape and size of plates and reduce the risk after surgery [72,73].

Careful neurological assessment is required as the clavicle is close to the apical pleura, brachial plexus, and subclavian artery and vein to exclude neurological deficits, pneumothorax, and distal perfusion problems [74]. In addition, these structures are at risk of injury from a fracture fragment or during fracture fixation [5,74].

It is important to emphasize that healthcare providers must assess each patient individually and consider various factors, such as age, activity level, and fracture type, when determining the most appropriate treatment and rehabilitation plan for midshaft clavicle fractures. The highest possible level of autonomy and quality of life for the patient is always the goal.

Several FE models have been developed in recent years to investigate bone stress shielding after fracture fixation, demonstrating that the FE model is a useful instrument for the analysis of biomechanics [10,75,76]. As shown in other studies [44,77,78], the limits of the studies with finite elements are that they are computational models and may not fully capture the complex biomechanical behavior of the clavicle and surrounding structures. Despite these limitations, FE studies have demonstrated that load distribution in a clavicle fracture treated with plates and screws differs from that of a healthy clavicle.

To provide better data on which to base treatment decisions, more randomized and prospective trials are needed. Ultimately, the treatment option must be chosen by the individual patient, carefully considering the relative benefits and risks of each intervention and the patient’s preferences.

## 5. Conclusions

FE simulation may be useful in predicting the biomechanical behavior of different implant configurations in plate fixation of midshaft clavicle fractures and may provide valuable insight into the optimal number, size, and position of screws to achieve maximum stability and functional outcomes. The stress distribution in a plate and screw fixation is a critical factor to consider when treating a clavicle fracture. This is influenced by several factors, including the position and design of the plate, the screw type and numbers used, and the biomechanical forces applied during shoulder elevation. The number and position of screws used in plate fixation can influence the rehabilitation plan, with a greater number of screws providing greater stability and potentially allowing an earlier range of motion exercises. The results showed that the stresses on the fixed clavicle are supported by the plate and screws in the most common type of solicitation, highlighting the importance of the screws in absorbing and distributing loading forces and in the healing process. Overall, the decision between operative and non-operative management of midshaft clavicle fractures should be made on a case-by-case basis, considering the specific needs and characteristics of the patient, as well as the degree of displacement and potential complications associated with each treatment option.

## Figures and Tables

**Table 1:** Finite element model parts.

Body Components	Elements	Nodes
Rib cage	12.361	13.526
Humerus	18.452	21.458
Scapula	17.232	19.557
Acromion	10.784	15.420
Coracoacromial ligament	5.469	5.914
Glenohumeral ligament	4.752	5.112
Acromioclavicular ligament	2.845	2.946
Coracohumeral ligament	3.025	3.412
Transverse humeral ligament	5.231	5.417
Joint capsule	4.569	4.822

**Table 2:** Equivalent von Mises maximum stresses and percentage difference localized in model parts.

Body Components	Equivalent V. Mises Stress [MPa]
Humerus	40
Scapula	30
Clavicle	9
Fractured clavicle	15
Coracoacromial ligament	8
Glenohumeral ligament	11
Acromioclavicular ligament	10
Coracohumeral ligament	13
Transverse humeral ligament	8
Joint capsule	8

## Data Availability

Data are contained within the article.
